# Some Causes of Cancer

**Published:** 1889-01

**Authors:** 


### SOME CAUSES OF CANCER.

Whether this terrible disease be constitutional or not in its origin, there can be no question that the determining cause of its appearance is in very many cases an injury (as a blow), or a condition resulting from an injury (as a scar), or the persistent application to a particular spot of something that keeps the tissue inflamed and "angry" (such as a jagged tooth which chafes the tongue). Workers in paraffine and petroleum are peculiary liable to cancer of the parts which are habitually exposed to the action of these substances. When soot commanded a good price, it had to be sifted ; and "chimney sweep's cancer" was a frequent result. Now-a-days it does not pay to sift the soot, and the disease to which it gave rise has disappeared.

Among causes of local irritation, heat is certainly one of the most active. By far the most common seat of malignant disease in men is the mouth, which is more exposed than any part of the body to irritation by hot substances. Every surgeon is familiar with this fact. Whether it be a lower lip on which the hot stem of a clay pipe or the smouldering paper of a cigarette has rested day after day ; or a tongue exasperated by the frequent contact of acrid tobacco smoke, or the mouth-piece of a foul pipe, or made raw by ardent liquors, or stung and blistered by fiery condiments, the cause is essentially the same, viz: the searing or irritation of the superficial covering by prolonged heat or pungent impressions. In Cashmir, where hot braziers are often applied to the abdomen and thigh, cancer of the parts is not uncommon, though all but unknown in either of these situations elsewhere.

It is highly probable that, in addition to the local irritation, some particular predisposition must exist in the patient, though in what this consists we do not know. That in a large number of cases the tendency to cancer is hereditary, there can be no question.—*Mackenzie's "Fatal Illness of Frederick the Noble."*

